# Operating Together: Challenges and Solutions for Sustaining Impactful Global Health Partnerships From 18 years of the RCSI/COSECSA Surgical Training Collaboration

**DOI:** 10.1002/wjs.70273

**Published:** 2026-02-23

**Authors:** Eric O'Flynn, Jane Odubu Fualal, Declan Magee, Wakisa Mulwafu, Lucia Brocato, Abebe Bekele, James Geraghty, Godfrey Sama Philipo, Eric Borgstein, Juan Carlos Puyana, Laston Chikoya

**Affiliations:** ^1^ Royal College of Surgeons in Ireland Dublin Ireland; ^2^ College of Surgeons of East, Central and Southern Africa Arusha Tanzania; ^3^ Department of Surgery Mulago National Referral Hospital Kampala Uganda; ^4^ Department of Surgery Kamuzu University of Health Sciences Blantyre Malawi; ^5^ University of Global Health Equity Kigali Rwanda; ^6^ St Vincent's University Hospital Dublin Ireland; ^7^ Kamuzu University of Health Sciences Blantyre Malawi; ^8^ Department of Surgery University of Pittsburgh Pittsburgh Pennsylvania USA; ^9^ Levy Mwanawasa Medical University Lusaka Zambia

**Keywords:** education, global surgery, partnership, training

## Abstract

**Background:**

International support for surgery and healthcare in low‐resource settings is primarily channeled through partnerships. To be truly impactful, such partnerships must endure long enough to mature, however longevity appears to be rare. Analysis of the challenges faced by successful long‐term partnerships and how they were overcome may offer useful lessons for newer and aspiring global health partnerships.

**Methods:**

The surgical training collaboration between the Royal College of Surgeons in Ireland and the College of Surgeons of East, Central, and Southern Africa has continued for 18 years and has delivered significant benefits for both partner institutions. Challenges faced by the collaboration and solutions to these challenges were elicited from key stakeholders in each college through an inductive approach.

**Results:**

Challenges and solutions reported were grouped under four domains: power, operational capacity, changing needs, and maximizing impact. A set of governance structures are proposed to mitigate power disparities between partners and between individuals. Leveraging nonclinical staff members to support development of back‐office systems increases local operational capacity to effectively engage in partnership activities. Constant change is a challenge for partnerships, which must both be accepted and planned for. The impact of work done through partnerships can be multiplied by expanding the collaboration to other comparable or synergistic institutions and making resources available open access.

**Conclusions:**

The RCSI/COSECSA collaboration program demonstrates that, over time, global health partnerships can play a transformational role in improving health outcomes in low‐resource settings, while also benefitting high‐income partners. Analysis of the development of the collaboration offers practical strategies for the development of other global health partnerships.

## 1 Introduction

Global Health Partnerships (GHPs) are at the heart of global surgery and global health more generally. Although GHPs can take many forms, a common model is a bilateral partnership between an institution in a low‐resource setting and an institution in a high‐resource setting [1], and it is in describing such “North‐South” partnerships that the term “global surgery” is most often used [2]. Many case studies of GHPs report impressive outcomes [3], although some evidence may be lacking in quantity and rigor [4]. Where the attributes of successful GHPs are identified, the need for long‐term partnership sustainability is a recurring theme [5, 6, 7] as time is required for GHPs to “mature to the transformational level” [6]. Guilfoyle, Morzycki, and Saleh consider that “meaningful impactful change takes time, often measured in decades” [7]. Longevity unfortunately appears to be an aspiration rather than a reality for most GHPs. Drawing on our 18 years of experience of collaboration between the College of Surgeons of East, Central, and Southern Africa (COSECSA) and the Royal College of Surgeons in Ireland (RCSI), we identify challenges that we believe are common to many GHPs and solutions that they can implement to achieve greater sustainability and impact.

### 1.1 COSECSA

**FIGURE 1:**
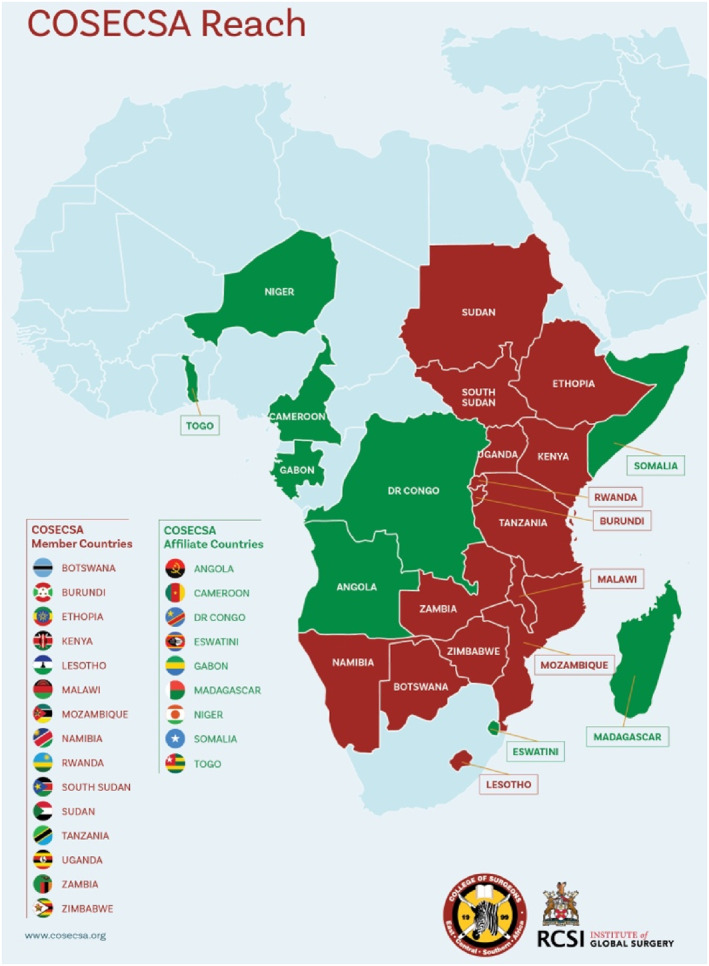
COSECSA member countries and affiliate countries.

The vast majority of people in the East, Central, and Southern Africa (ECSA) region lack access to life‐saving emergency surgical care when needed [8, 9] and for those who do receive surgery, outcomes are worse than the global average [10]. An insufficient number of surgical care providers is a key barrier to the provision of more and safer surgery. Most ECSA countries have fewer than 1 surgeon per 100,000 population [11] and fewer than 1 anesthesiologist per 200,000 population [12], far below international minimum recommendations [9, 13]. COSECSA was founded in 1999 by the Association of Surgeons of East Africa to train surgeons for the ECSA region. Prior to the advent of COSECSA, surgical training in the region had been undertaken in a limited number of programs in university teaching hospitals with constrained trainee numbers. COSECSA formulated a common postgraduate surgical training program, undertaken in accredited training hospitals with a common exam and a regionally recognized qualification. Starting with eight member countries, COSECSA now encompasses 15 countries, as shown in Figure 1, with a combined population of approximately 547 million [14]. There are also COSECSA accredited training hospitals in nine additional COSECSA affiliate countries.

### 1.2 RCSI

RCSI was founded in 1784 as the national training body for surgery in Ireland. RCSI today is both an internationally focused university of medicine and health sciences with campuses in Ireland, Malaysia, and Bahrain, and the national postgraduate training body for surgery and related specialties in Ireland.

## 2 Methods

### 2.1 Establishing the Collaboration

The simply named “RCSI/COSECSA Collaboration Program” (hereafter “the Collaboration”) was founded in 2007, with both colleges stating that they “wish to work together to improve the standards of surgical care in the region of East, Central and Southern Africa by advancing surgical education, training and examinations” [15]. Believing that local institutions are best placed to respond to local needs, the Collaboration takes an “institutional capacity building” approach [16], through which institutions “obtain, strengthen and maintain the capabilities to set and achieve their own development objectives” [17]. This way of working has been described as “accompaniment… sticking with a task until it's deemed completed by the person or people being accompanied, rather than by the *accompagnateur*” [18]. The collaboration has been consistently financially supported by Irish Aid, Ireland's development cooperation program, since 2008, with over €6m funding provided. This investment has been more than matched by the significant in‐kind contributions provided by both partner organizations.

### 2.2 Collaboration Activities and Outputs

The collaboration is a “whole college” collaboration, involving a wide range of staff and faculty in both institutions, and a diverse range of activities related to improving and expanding COSECSA examinations, training, data collection, research, online resources, advocacy, quality assurance, and administrative capacity. Selected outputs are shown in Table 1. As well as supporting these key functions, the collaboration has equally supported COSECSA's capacity to deliver them independently, through provision of finance, support for staffing costs and staff development, strategic planning facilitation, and the codevelopment of administrative tools and processes.

**TABLE 1:** Selected outputs of the RCSI/COSECSA collaboration.

Faculty development	Training of trainers, master trainers and surgical basic science faculty
Educational tools	Development of bespoke e‐learning platform and electronic training logbook
Examinations	Development of online examiner course and other tools, transition of examination format, examiner exchanges, and psychometric analysis
Research	Provision of research grants, mentorship, methodology training, journal support, data collection, and analysis expertise
Leadership	Online clinical leadership courses for training program directors
Capacity building	Strategic planning, staffing support, and codevelopment of administrative systems
Training other cadres	1000+ nonspecialist doctors trained in essential surgical skills

### 2.3 Methodological Approach

Challenges faced by the collaboration and their solutions were elicited from the authorship group through a series of unstructured discussions with all authors, in some cases individually, in other cases collectively. Shorthand notes were taken which were analyzed through a general inductive approach [19]. Elicited challenges and solutions were synthesized and results iteratively refined until consensus was achieved. The authorship group are all current or past collaboration steering committee members or currently or previously employed to work on collaboration activities.

## 3 Results

Over 18 years, we have identified challenges in our partnership which we believe are common to many GHPs. We have also identified solutions, enabling us to sustain our partnership over the long term. These solutions may similarly be applicable to many GHPs. We do not directly discuss challenges related to financing GHPs as we do not consider there is a universally applicable solution—different partnerships are funded in different ways.

### 3.1 Domain 1—Power

#### 3.1.1 Challenge

Power dynamics are a challenge for most GHPs. Several factors may be at play. High‐resource setting partners are often either the source, or conduit, of funding for the low‐resource setting partner. COSECSA's Francis Omaswa described how this dynamic has often led African institutions to implement solutions that they knew would not work, in exchange for funding [20]. Unhelpful, often unquestioned, received ideas can influence power dynamics, such as an “assumed expertise gradient” [21], where expertise is assumed to flow from the high‐resource setting partner to the low‐resource setting partner. Particular individuals—from both high‐ and low‐resource setting partners—may exert disproportionate influence on the GHP. Often those individuals with the passion to create the partnership, maintain an unhelpfully strong influence over it, and ultimately unwittingly constrain the growth of the partnership. This challenge is known as “founder's syndrome” [22]. High‐resource setting partners often have other advantages which may not be available to their partner, such as staff time, that can be assigned to work on collaborative activities.

#### 3.1.2 Solution

Formal governance mitigates these challenges. The collaboration was quickly embedded in both colleges as an institutional partnership with formal governance arrangements. A steering committee with six members from each college meets at least three times a year, in meetings alternately chaired by each college. New programs of work are developed through a series of workshops. Responsibility for managing the Collaboration budget is shared. The budget for each activity is managed by the college which is leading on implementation. Funding is allocated by COSECSA for the staff time required for collaboration activities, enabling COSECSA to increase its staff numbers and capacity. Grant agreements and a reporting framework ensure financial accountability. These structures ensure that each College's strengths, challenges, and perspectives are embraced in decision‐making processes.

Demonstrating commitment to the partnership and shared goals has helped strengthen institutional connections. A notable example has been the examiner exchange program, in which examiners from each college examine in the other's exams. This has been both a bidirectional exchange of knowledge and best practice and a mark of respect. Both colleges have formally recognized important individual contributions to the collaboration. Former presidents of COSECSA, Professor Krikor Erzingatsian and Professor Francis Omaswa, were awarded honorary fellowships of RCSI, and RCSI gifted COSECSA their chain of office. As well as admitting RCSI Fellows by election and awarding honorary fellowships to RCSI recipients, COSECSA awards the highest scoring candidate in its examinations the “Professor Gerald O'Sullivan” prize, named for the former RCSI president. A fellowship grant from RCSI named after RCSI's Professor Sean Tierney is available to recent COSECSA graduates.

**FIGURE 2:**
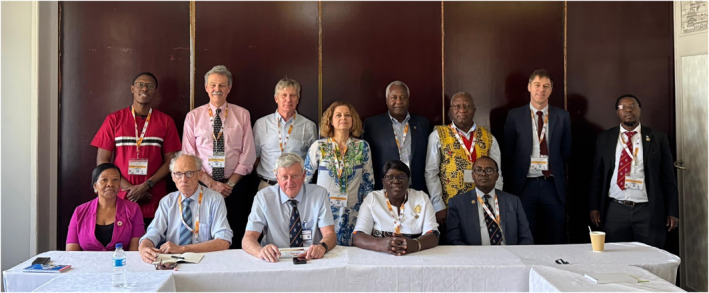
RCSI/COSECSA steering committee meeting, Harare, Zimbabwe, December 2024. Standing, L‐R: Michael Mwachiro, Juan Carlos Puyana, Kieran O'Driscoll, Lucia Brocato, Samwel Nungu, Celestine Mbangtang, Eric O'Flynn, Wakisa Mulwafu. Seated, L‐R: Stella Itungu, Eric Borgstein, James Geraghty, Jane Fualal Odubu, and Laston Chikoya.

The founders of the Collaboration in 2007 were COSECSA's Professor Krikor Erzingatsian and RCSI's Professor Gerald O'Sullivan. Unfortunately, neither Professor Erzingatsian nor Professor O'Sullivan are alive to see what has been achieved. Indeed no original member from 2007 remains on the steering committee; the Collaboration continues to draw new collaborators and new energy from both colleges and from new partner institutions. Steering committee members are shown in Figure 2.

### 3.2 Domain 2—Operational Capacity

#### 3.2.1 Challenge

For most low‐resource setting partners in GHPs, it is not only academic or clinical expertise which is required but also a wide range of management, administrative, and technical skills. These are often costly or impossible to obtain in the open market.

#### 3.2.2 Solution

As an institutional capacity building program, the collaboration can leverage RCSI faculty, staff, and intellectual property for COSECSA's benefit. This nonacademic support has encompassed everything from RCSI senior management facilitation of COSECSA strategic planning sessions to RCSI's design department adding a professional design touch to key COSECSA documents. RCSI departments as diverse as finance, psychometrics, communications, IT, surgical training, simulation, healthcare management, and population health contribute to collaboration activities. RCSI staff and faculty report personal and professional learning and growth from these engagements.

RCSI IT and e‐learning professionals, in particular, have made a substantial contribution to the collaboration, producing electronic tools to facilitate management, quality assurance, and the delivery of a common curriculum over the vast geographic area covered by COSECSA. Bespoke electronic training logbooks, available on desktop and mobile apps, facilitate live oversight of COSECSA trainees [23]. The COSECSA e‐learning platform hosts over 70 courses, with content variously produced by the Collaboration, by other partners, and independently by COSECSA. A database of surgeons in the region was collaboratively built and is maintained by COSECSA as part of its day‐to‐day operations. Bespoke training management software simplifies admissions and financial processes, and an online system was built to manage the research ethical approval process. These developments have enabled datasets of international importance to be collected. RCSI technicians and researchers have supported COSECSA data collection and analysis, which has in turn supported and informed COSECSA's growth. The COSECSA logbook contains over 700,000 recorded procedures as of November 2025, providing important insight into training and practice in the region [23, 24, 25, 26, 27, 28]. The COSECSA database has been a key input to studies describing the surgical workforce in the region [11, 29, 30, 31].

We believe that the nonclinical staff of high‐resource setting partners are an underused resource available to many GHPs.

### 3.3 Domain 3—Changing Needs

#### 3.3.1 Challenge

As partners' needs change and their capacity grows, so must the aims and activities of their partnerships. An effective partnership is necessarily project based. Partnerships should not permanently take on day‐to‐day operational activities that could be managed by either partner. This all means that effective GHPs are constantly changing.

#### 3.3.2 Solution

In the collaboration, we can see this constant evolution. Broadly speaking, in the period 2007–2011, the collaboration focused on supporting the establishment of quality exams and training. In 2012–2016, there was a significant focus on developing local training capacity. From 2017 to 2020, emphasis was placed on developing management and administrative systems to manage increasingly large and complex training programs. From 2021 to present, a key development has been to support training of all members of the surgical team—recognizing that surgeons are a necessary but insufficient part of the surgical care workforce.

**FIGURE 3:**
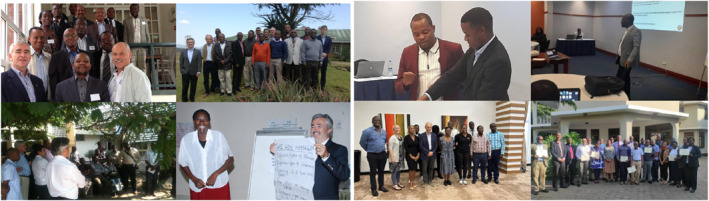
Train the trainer courses with RCSI (left) and COSECSA run (right) courses.

Although the collaboration is long‐term, COSECSA is and always has been an independent organization. COSECSA's capacity to achieve its aims is ever‐growing and Collaboration activities have been “handed over” as COSECSA capacity increased. An illustrative example can be seen in the training of surgeons as medical educators. This program started in 2010 [32] with numerous train the trainer programs conducted by RCSI master trainers until 2014. In 2014 and 2015, COSECSA cohorts of master trainers were trained by RCSI to empower these local faculty to deliver the train the trainer program. These courses continue to be run independently by COSECSA. As of May 2025, 904 COSECSA surgeons have been trained as trainers and 77 as master trainers. RCSI facilitated courses and COSECSA‐led courses are shown in Figure 3.

Future collaborative activities will be based on COSECSA's needs. Indeed, at a future point, should there not be a productive role for the collaboration to play, it will not represent a failure of the collaboration but rather stand as a testament to its success.

### 3.4 Domain 4—Maximizing Impact

#### 3.4.1 Challenge

High‐functioning GHPs develop expertise and generate new knowledge, tools, and processes. Many of these outputs may be applicable to other partnerships and institutions; yet, apart from academic publications, there may not be a clear pathway by which they can be shared.

#### 3.4.2 Solution

GHPs should spread the benefits of partnership beyond the original intended partner institutions through the inclusion of more partner institutions or by making outputs of the GHP available open access.

The collaboration program has done both. Understanding that surgeons are a necessary but insufficient part of the multidisciplinary surgical team, RCSI and COSECSA expanded the collaboration to support other ECSA colleges. The College of Anesthesiologists of East, Central, and Southern Africa (CANECSA) joined in 2019, with the East, Central, and Southern Africa College of Nursing and Midwifery (ECSACONM) and the East, Central, and Southern Africa College of Obstetrics and Gynecology (ECSACOG) both joining in 2020. The College of Anesthesiologists of Ireland, the RCSI Faculty of Nursing, and the Institute of Obstetricians and Gynecologists in the Royal College of Physicians in Ireland joined to bring specialist expertise from Irish institutions. Most collaboration activities in which these newer partner organizations have been involved draw heavily upon similar activities previously undertaken by COSECSA and RCSI. These include.

- Mapping the regional obstetrician/gynecologist and anesthesiologist [12] workforces, using the same methodology as has been employed to map the surgical workforce [11, 29].
- Launching the CANECSA e‐learning platform, built on the same foundations as COSECSA's platform.
- Developing the CANECSA electronic logbook and administrative systems, both of which are similar to COSECSA versions.
- Development of a perioperative nursing e‐learning course [33] using a methodology developed for creation of surgical e‐learning content.
- Incorporating nurses, obstetricians, and anesthesiologists into COSECSA train the trainer programs, and launching independent CANECSA train the trainer and master trainer programs.

**FIGURE 4:**
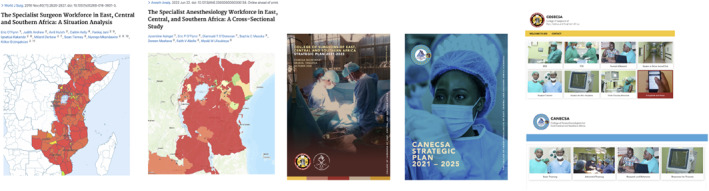
Not reinventing the wheel: COSECSA and CANECSA workforce mapping research, strategic plans, and E‐learning platforms.

Examples of similar collaborative work undertaken with both COSECSA and CANECSA are shown in Figure 4.

COSECSA and the other ECSA colleges, independently and in partnership, have developed resources that can be used in developing training programs outside the region, and a training model that can be emulated. Notably, e‐learning courses developed by COSECSA and CANECSA and supported by the collaboration as well as a course co‐developed by ECSACONM and the RCSI Faculty of Nursing and Midwifery have been made available open‐access to the world on the United Nations Global Surgery E‐Learning Hub [34] and translated into other languages due to global demand.

## 4 Discussion

Measuring the impact of any GHP can be complex as it is often difficult to separate the achievements of the partnership from those of the partner institutions themselves. For GHPs, which take a capacity building approach and are not involved in the direct provision of care to patients, the difficulty in quantifying impact may be even greater. However, it is possible to observe significant growth and development in the partner organizations during the period of the RCSI/COSECSA collaboration. It is reasonable to consider that the collaboration has played a meaningful role in enabling and accelerating this progress. The collaboration uses theory of change [35] methodology to explain the means by which the collaboration enables these impacts and changes.

### 4.1 Impact of the Collaboration on COSECSA

**FIGURE 5:**
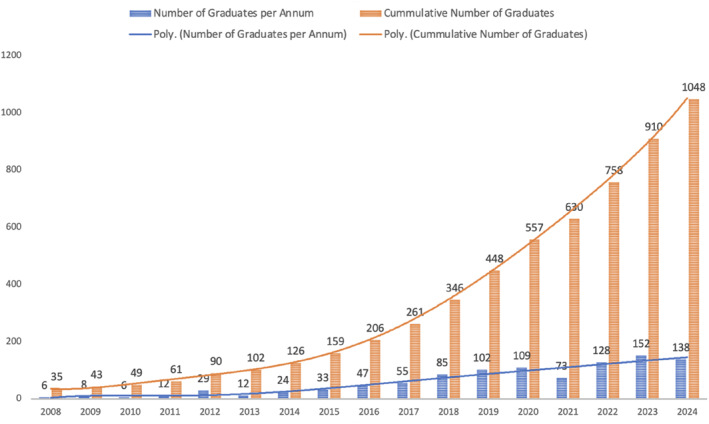
COSECSA graduates by year.

The growth and transformation of COSECSA since the inception of the Collaboration has been remarkable by any measure. COSECSA is now playing a pivotal role developing the surgical workforce in East, Central, and Southern Africa [36], having surpassed its strategic targets of graduating 500 new surgeons by 2020 and 1000 by 2025 [37] as shown in Figure 5. To put this achievement in context, in 2015, there were just 1690 surgeons in total in the then nine COSECSA countries [29]. In order to improve and expand surgical services, it is vital that growth in the surgical workforce significantly outstrips the rapid population growth in the region.

We recognize that many factors other than the total number of surgeons affect the availability of surgery, including the availability of other members of the surgical team, infrastructure, consumables, and funding. Although also recognizing that factors other than the influence of COSECSA affect the surgeon:population ratio—including graduates of other training programs, retirement and migration of the current workforce, and population growth—we can nevertheless estimate the impact of COSECSA graduates on the availability of surgery in the region.

Each new COSECSA surgeon can be expected to perform 300 procedures per year. In a context where baseline surgical volume may be as low as 212 procedures per 100,000 population across the continent [10], the procedures performed each year by each new surgeon is equivalent to a doubling of surgical volume for a population of 141,509 people. Therefore, the procedures performed by the 1048 COSECSA graduates are equivalent to a doubling of the volume of surgery available to a population of over 148 million people.

### 4.2 Impact of the Collaboration on RCSI

From the foundations of the Collaboration, RCSI developed significant new global surgery research and educational programs and partnerships, a number of which have been in collaboration with COSECSA [38]. These expanded activities ultimately led to the foundation of the RCSI Institute of Global Surgery in 2021 [39]. The Institute of Global Surgery is dedicated to expanding access to quality surgical care in low‐resource settings and now employs 13 staff, as well as faculty and scholars, working on projects around the world, many of which involve COSECSA. The collaboration has enhanced RCSI's international reputation. Among other awards, the collaboration won the 2025 AMEE ASPIRE‐to‐Excellence Award for International Collaboration in Health Professions Education [40]. It is perhaps not unrelated to RCSI's work in global surgery that RCSI is ranked first among universities globally for contribution to UN Sustainable Development Goal 3 ‘Good Health and Wellbeing’ in the Times Higher Education University Impact Rankings [41]. The Collaboration has contributed to RCSI staff development; over 150 RCSI staff and faculty have directly worked on Collaboration activities. RCSI staff members report important that learning from this work has benefitted RCSI. For example, the collaboration supported the foundation of the COSECSA Women in Surgery Africa [42] group in 2015 to support women in surgery and surgical training. The RCSI lead individual on this activity subsequently went on to manage the production of the RCSI PROGRESS Women in Surgery report [43], explicitly bringing this learning from COSECSA to RCSI.

### 4.3 Impact of the Collaboration on Other African Training Colleges and Beyond

ECSA collaboration partner colleges have had many notable achievements since joining the collaboration—some of which had little collaboration involvement, whereas in others, the collaboration played a key role. CANECSA and ECSACOG launched collegiate anesthesiology and obstetric/gynecological training in 2021 and 2022, respectively. ECSACONM launched a perioperative nurse training program in 2022, developed through the collaboration. Irish partner organizations have continued to grow their global health involvement, IOG obtained external grant support to develop its relationship with ECSACOG whereas CAI has set up a global health committee, employed a global health administrator and mobilized volunteers.

## 5 Conclusion

GHPs can play a transformational role in improving health outcomes worldwide, but require time to do so. The RCSI/COSECSA Collaboration Program has had a profound long‐term impact on surgical training in East, Central, and Southern Africa as well as an important impact on RCSI and all other partner organizations. Lessons can be drawn from the challenges faced and solutions adopted by the Collaboration to inform GHPs looking to achieve longevity and long‐term impact.

## Author Contributions

**Eric O'Flynn:** conceptualization, project administration, data curation, formal analysis, writing – original draft, writing – review and editing. **Jane Odubu Fualal:** conceptualization, writing – original draft, writing – review and editing. **Declan Magee:** conceptualization, writing – original draft, writing – review and editing. **Wakisa Mulwafu:** conceptualization, writing – original draft, writing – review and editing. **Lucia Brocato:** conceptualization, writing – original draft, writing – review and editing. **Abebe Bekele:** conceptualization, writing – original draft, writing – review and editing. **James Geraghty:** conceptualization, writing – original draft, writing – review and editing. **Godfrey Sama Philipo:** conceptualization, writing – original draft, writing – review and editing. **Eric Borgstein:** conceptualization, writing – original draft, writing – review and editing. **Juan Carlos Puyana:** conceptualization, writing – original draft, writing – review and editing, supervision. **Laston Chikoya:** conceptualization, writing – original draft, writing – review and editing, supervision.

## Funding

The RCSI/COSECSA Collaboration Program is funded by Irish Aid, the Irish Government's official development aid program. No specific funding was received for this research.

## Ethics Statement

Data provided in this article are available in the public domain or is nonsensitive educational research which is unlikely to adversely impact learners, and thus does not require research ethics review.

## Conflicts of Interest

The authors declare no conflicts of interest.

## Data Availability

Data sharing is not applicable to this article—background information about RCSI, COSECSA, and the RCSI/COSECSA Collaboration Program is available in the public domain, whereas the summarized qualitative research is included in the published article.
